# Disseminated Fungal Infection in Profoundly Immunocompromised Hosts: A Three-Case Series

**DOI:** 10.7759/cureus.104022

**Published:** 2026-02-21

**Authors:** Alan Wang, John Greene

**Affiliations:** 1 Osteopathic Medicine, Nova Southeastern University Dr. Kiran C. Patel College of Osteopathic Medicine, Clearwater, USA; 2 Infectious Diseases, Moffitt Cancer Center, Tampa, USA

**Keywords:** disseminated fungal infection, fusarium species, immunocompromised, neutropenia, opportunistic fungal infection, scedosporium prolificans

## Abstract

Disseminated fungal infection (DFI) is uncommon in the general population but represents a severe and frequently fatal complication in immunocompromised patients, particularly those with hematologic malignancies and prolonged neutropenia. In high-risk settings, invasive fungal infections occur with clinically meaningful frequency; a large systematic review of critically ill patients reported a pooled prevalence of invasive fungal infection of approximately 5%, highlighting that while DFI is uncommon in the general population, serious fungal disease is encountered more frequently in select high-risk cohorts. We report a case series of three immunocompromised patients with DFI to highlight shared clinical patterns, diagnostic limitations, and poor outcomes. Two patients developed disseminated fusariosis with pulmonary nodules and characteristic necrotic cutaneous lesions, while the third developed *Scedosporium prolificans* fungemia with pulmonary involvement and progressive encephalopathy concerning for early central nervous system (CNS) dissemination. Diagnosis required a multimodal approach, including tissue biopsy, bronchoalveolar lavage (BAL), blood cultures, and advanced molecular testing; however, precise quantification of diagnostic delay was not systematically available across cases. In this series, diagnosis was nonetheless delayed relative to initial clinical presentation, largely due to nonspecific radiographic findings, initially negative or indeterminate cultures, overlap with bacterial or noninfectious etiologies, and the need for invasive sampling in profoundly cytopenic patients. Management was further limited by intrinsic antifungal resistance, drug toxicity, and failure of immune recovery; despite aggressive therapy, all patients experienced rapid clinical deterioration over days to weeks following diagnosis, culminating in death or transition to hospice care. These cases underscore the aggressive nature of DFI in profoundly immunocompromised hosts, the central role of neutropenia in facilitating dissemination, and the need for early recognition, multidisciplinary management, and timely goals-of-care discussions when DFI occurs in the setting of advanced immunosuppression.

## Introduction

Disseminated fungal infection (DFI) represents a severe and frequently fatal complication in immunocompromised hosts, particularly among patients with hematologic malignancies, prolonged neutropenia, or exposure to cytotoxic chemotherapy. While DFI is uncommon in the general population, it occurs with clinically meaningful frequency in select high-risk cohorts, including patients with hematologic malignancies and critical illness. Globally, updated modeled estimates from The Lancet Infectious Diseases (2024) suggest an annual incidence of approximately 6.5 million invasive fungal infections and 3.8 million associated deaths, of which 2.5 million are estimated to be directly attributable to invasive fungal disease [1], with disseminated disease affecting an estimated 1.9 million people worldwide [2]. In this context, disseminated disease refers to fungal infection involving two or more noncontiguous organ systems, typically resulting from hematogenous spread beyond the initial portal of entry. It is associated with the highest morbidity and mortality due to rapid hematogenous spread, diagnostic delays, and limited therapeutic options. Although *Candida*, *Aspergillus*, and *Cryptococcus* species account for the majority of disseminated fungal disease worldwide, rare and intrinsically resistant molds such as *Fusarium* spp. and *Scedosporium/Lomentospora* spp. are increasingly recognized as important causes of DFI in profoundly immunosuppressed patients [2-7].

Host defenses and immunogenetic factors play critical roles in determining the manifestations and severity of fungal disease. For example, pulmonary aspergillosis may present as a chronic process in otherwise immunocompetent individuals with underlying structural lung disease, whereas immunocompromised hosts may develop rapidly progressive, angioinvasive pneumonia [3]. Angioinvasion, defined as fungal invasion of blood vessel walls leading to thrombosis, tissue ischemia, and necrosis, is a hallmark of severe disseminated mold infections and a key mechanism underlying multiorgan involvement. DFI typically arises when host immune defenses, including neutrophil-mediated phagocytosis and oxidative burst and T-cell-mediated adaptive immune responses, fail to contain fungal invasion at the portal of entry, most commonly the respiratory tract or skin. Infections caused by *Fusarium* spp. and *Scedosporium/Lomentospora prolificans* are generally acquired via inhalation of conidia or direct inoculation following trauma [4,5]. Once host defenses are impaired, angioinvasion facilitates hematogenous dissemination to distant organs, including the lungs, skin, central nervous system (CNS), spleen, kidneys, and eyes.

Clinical manifestations of DFI are often multisystemic. Pulmonary involvement commonly presents radiographically as nodules, consolidations, or infarct-like lesions, reflecting vascular invasion and tissue ischemia. Cutaneous disease is a hallmark of disseminated mold infection and frequently serves as a key diagnostic clue; up to 70% of patients with disseminated fusariosis develop painful erythematous or violaceous nodules with central necrosis, corresponding to angioinvasion and microthrombus formation; this estimate is derived primarily from retrospective cohort studies and case series conducted in hematologic and transplant populations and may be influenced by selection and temporal bias inherent to older, pre-mold-active prophylaxis eras [8,9]. Hematogenous spread may also result in metastatic involvement of organs. In candidemia, for example, endogenous fungal endophthalmitis occurs in < 2-20% of cases, depending on the population studied [3]. Disseminated *Scedosporium/Lomentospora* infections have been reported to involve the bloodstream, lungs, CNS, bones, joints, and soft tissues, with reported mortality rates approaching 87.5% in disseminated disease [6,7], compared with 50-70% in disseminated fusariosis [8,9].

The risk of DFI is driven by a complex interplay of host-, treatment-, and environment-related factors. Patients with acute leukemia, solid organ transplantation, or those receiving remission-induction chemotherapy are at particularly high risk due to prolonged and profound neutropenia, typically defined as an absolute neutrophil count (ANC) <500 cells/µL lasting more than 7 days or anticipated to persist for >7 days [3,5,10]. Additional risk factors include immunosuppressive therapies, underlying malignancy, and chronic inflammatory or ischemic tissue injury. Patients with anti-neutrophil cytoplasmic autoantibodies (ANCA)-associated vasculitis, for example, are at increased risk of DFI due to intensive immunosuppression, chronic pulmonary ischemic changes, and iron availability from alveolar hemorrhage, which may promote fungal growth [11]. Importantly, recovery from neutropenia has been independently associated with improved survival in patients with DFI [6], although experimental models suggest that fungal inoculum size also influences disease severity and outcome [5]. Despite advances in antifungal prophylaxis and diagnostics, breakthrough DFI may occur in patients receiving mold-active azole prophylaxis. Breakthrough infections have been reported in approximately 2-5% of high-risk patients receiving posaconazole or voriconazole prophylaxis, typically during prolonged courses extending beyond 7-14 days of neutropenia, particularly in the setting of profound or persistent immunosuppression [4,12]. Mortality remains high, particularly among infections caused by mold species with intrinsic antifungal resistance.

Here, we present three cases of DFI in immunocompromised patients, illustrating shared clinical patterns, diagnostic challenges, and poor outcomes across different fungal pathogens and immunosuppressive states.

## Case presentation

Case 1

**Figure 1:**
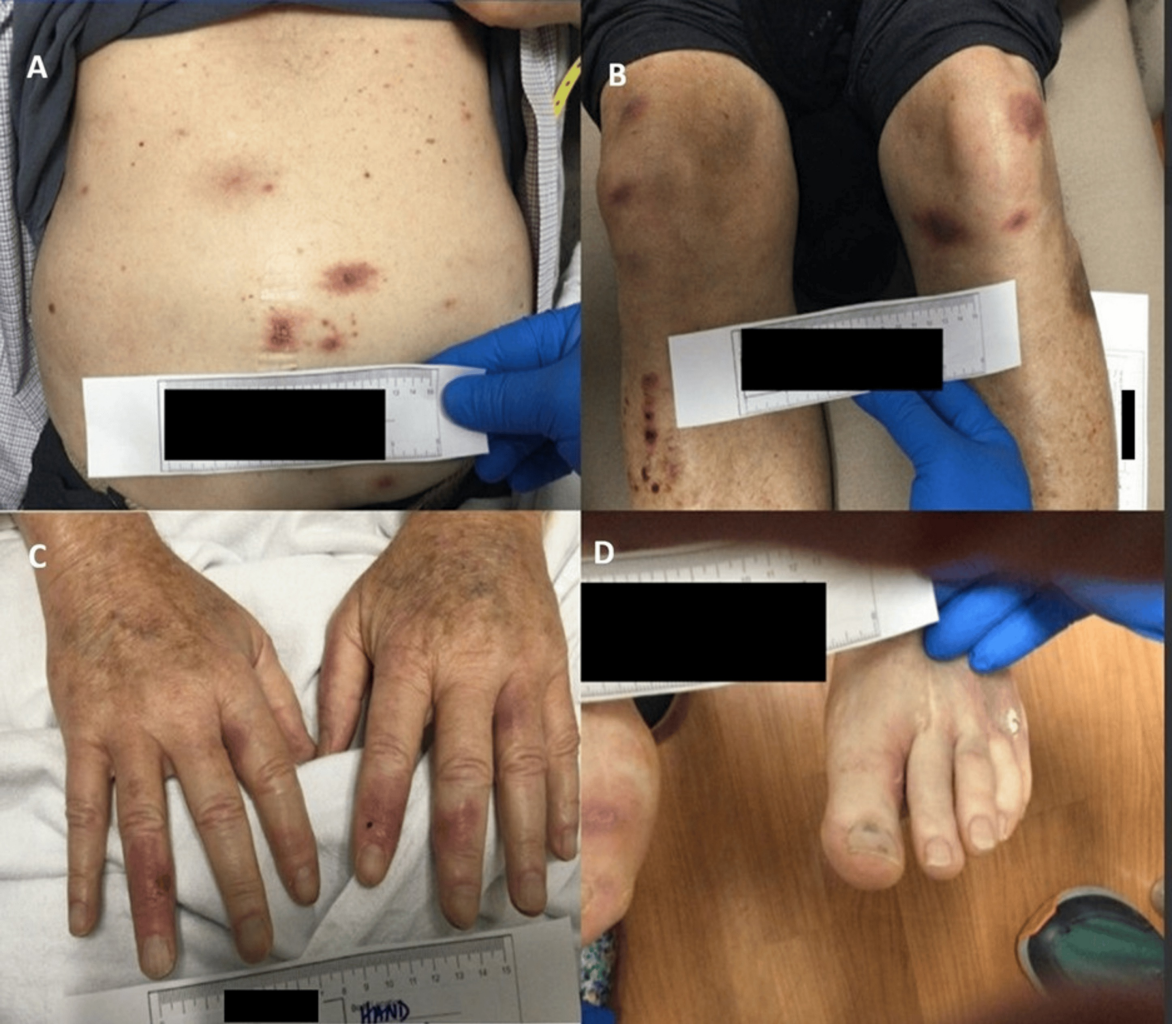
Cutaneous manifestations of disseminated fusariosis. Clinical photographs (A–D) showing multifocal erythematous to violaceous nodules and plaques with central necrosis; biopsy confirmed Fusarium species.

**Figure 2:**
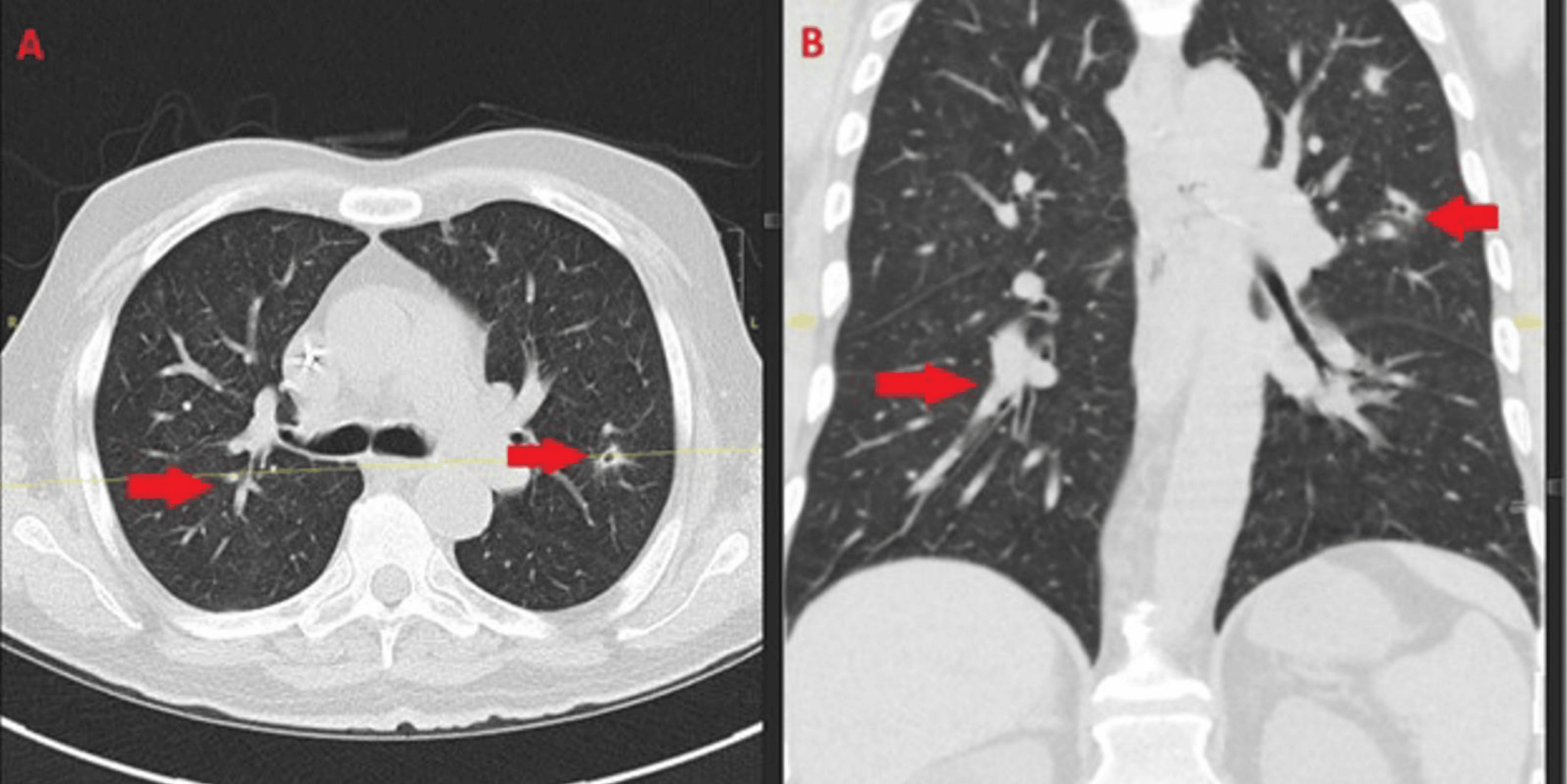
Pulmonary involvement in disseminated fusariosis. Axial (A) and coronal (B) chest CT images obtained on December 18, 2024, demonstrating bilateral peribronchovascular nodular and branching opacities (arrows) with surrounding ground-glass attenuation, suggestive of early halo-type changes. These findings are consistent with angioinvasive fungal pneumonia in a profoundly immunocompromised host with disseminated Fusarium infection. CT: computed tomography

A 75-year-old male with trisomy 8-associated de novo acute myeloid leukemia (AML), a baseline Eastern Cooperative Oncology Group (ECOG) performance status of 2, and comorbid hypertension and hyperlipidemia, without significant baseline cardiopulmonary or renal disease, was initiated on induction therapy with azacitidine and venetoclax. He achieved morphologic leukemia-free status (MLFS) after the first treatment cycle; minimal residual disease (MRD) assessment was not performed at that time. In October 2024, he established care at our institution for the consideration of allogeneic hematopoietic stem cell transplantation. He was admitted on December 18, 2024, for concern of DFI, presenting with diffuse erythematous cutaneous nodules involving the trunk and extremities that had been progressively developing over approximately 1-2 weeks prior to admission (Figure 1). These lesions had been biopsied at an outside hospital, with pathology positive for *Fusarium* species. A chest computed tomography (CT) performed on admission demonstrated bilateral peribronchovascular nodular and branching opacities with surrounding ground-glass attenuation, consistent with angioinvasive fungal pneumonia and suggestive of early halo-type changes related to hemorrhage and edema in the setting of disseminated fusariosis (Figure 2). A repeat skin biopsy at our institution again grew *Fusarium*, confirming disseminated disease.

**Figure 3:**
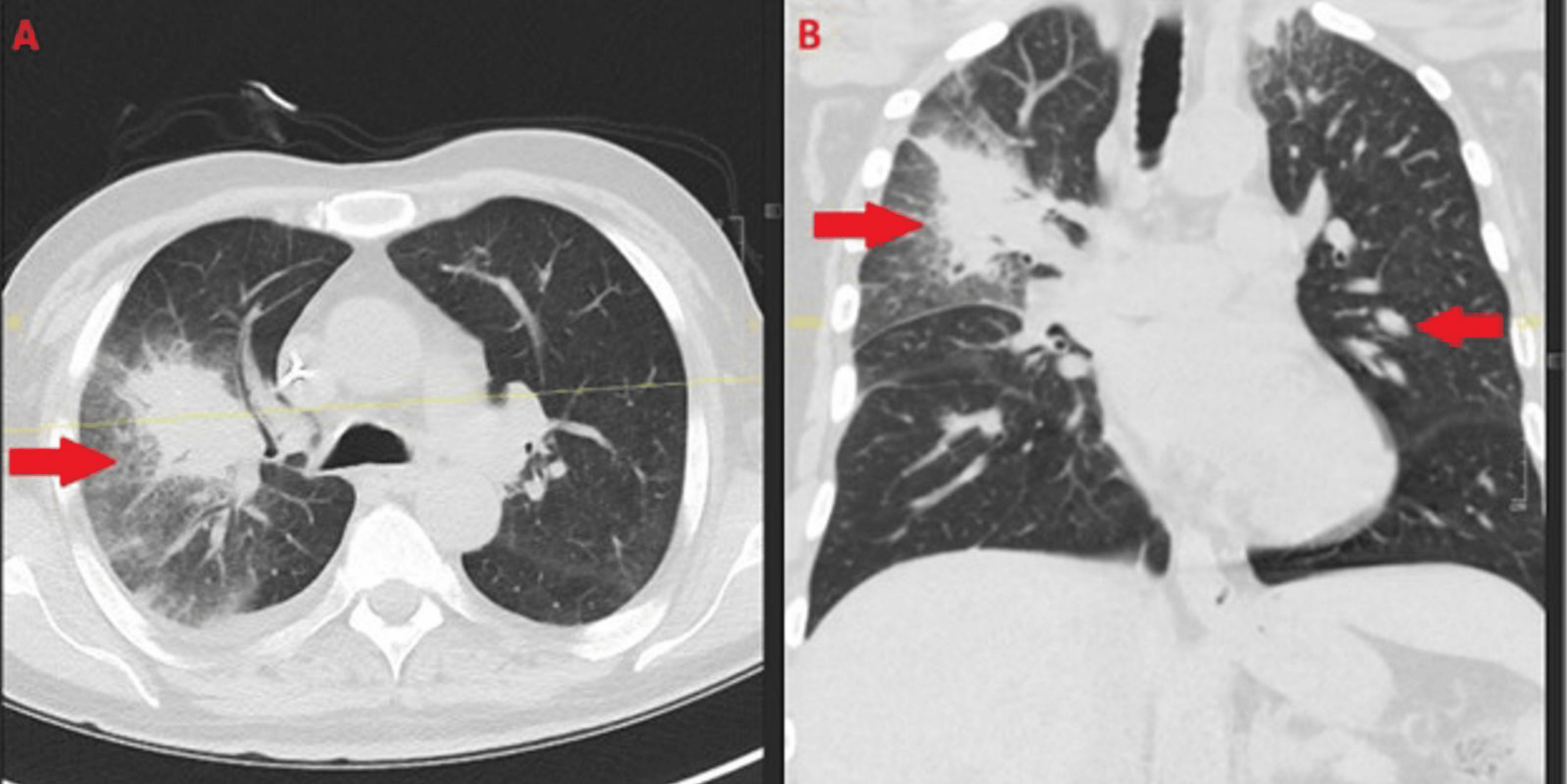
Interval progression of pulmonary fusariosis. Axial (A) and coronal (B) chest CT (January 9, 2025) showing worsening right upper lobe consolidation and persistent bilateral peribronchovascular opacities (arrows), consistent with progressive pulmonary involvement. CT: computed tomography

Infectious Diseases was consulted, and the patient was initiated on amphotericin B, voriconazole, and terbinafine, which were continued through January 8, 2025, while awaiting compassionate use approval for fosmanogepix. Fosmanogepix was approved and initiated on January 8, 2025, and the patient received four days of intravenous therapy, with a planned total treatment duration of six months. Given his profound immunocompromised state, levofloxacin and acyclovir prophylaxis were also initiated. A repeat chest CT obtained during hospitalization on January 9, 2025, demonstrated the interval progression of pulmonary findings concerning for worsening pulmonary fusariosis (Figure 3). However, no changes were made to his antimicrobial regimen at that time per the Infectious Diseases recommendations.

During hospitalization, a rapid response was activated for hypotension and dyspnea. The patient remained significantly debilitated, with laboratory evaluation notable for severe pancytopenia, including profound leukopenia with absolute neutropenia, marked anemia, and severe thrombocytopenia (Table 1). His neutropenia reached a nadir of zero and persisted without meaningful recovery for several weeks despite supportive measures, reflecting prolonged bone marrow aplasia in the setting of refractory leukemia and ongoing therapy. Given the severity of cytopenias, he required frequent transfusion support with packed red blood cells and platelets throughout hospitalization, with red blood cell transfusions administered to maintain hemoglobin above institutional thresholds (approximately 7 g/dL) and platelet transfusions given daily or more frequently to maintain platelet counts above bleeding-risk thresholds (<10,000-20,000 cells/µL). Laboratory studies also demonstrated hypogammaglobulinemia, with a markedly reduced immunoglobulin G level, consistent with impaired humoral immunity (Table 1). Intravenous immunoglobulin replacement was considered; however, it was not administered during this hospitalization due to the patient’s overall clinical trajectory and prioritization of infection-directed management. He received a dose of filgrastim in the setting of profound neutropenia. He was discharged on January 15, 2025, with close follow-up in Infectious Diseases and Hematology.

**Table 1:** Key laboratory findings at presentation. ↓: decreased; ↓↓: moderately decreased; ↑: increased ALT: alanine aminotransferase; AST: aspartate aminotransferase

Laboratory Parameter	Reference Range	Case 1	Interpretation	Case 2	Interpretation	Case 3	Interpretation
White blood cell count (cells/µL)	4,000–11,000	0	↓↓	200	↓↓	~200	↓↓
Absolute neutrophil count (cells/µL)	>1,500	0	↓↓	20	↓↓	~200	↓↓
Hemoglobin (g/dL)	13.5–17.5	5.8	↓↓	6–7	↓↓	7–8	↓
Platelet count (cells/µL)	150,000–400,000	2,000	↓↓	<20,000	↓↓	13,000	↓↓
Immunoglobulin G (mg/dL)	700–1,600	292	↓	Not available	-	Not available	-
Creatinine (mg/dL)	0.6–1.3	Normal	-	Normal	-	1.7–1.8	↑
AST/ALT (U/L)	<40/<40	Normal	-	Normal	-	Elevated	↑
Serum lactate (mmol/L)	0.5–2.2	Normal	-	Normal	-	Elevated	↑

**Figure 4:**
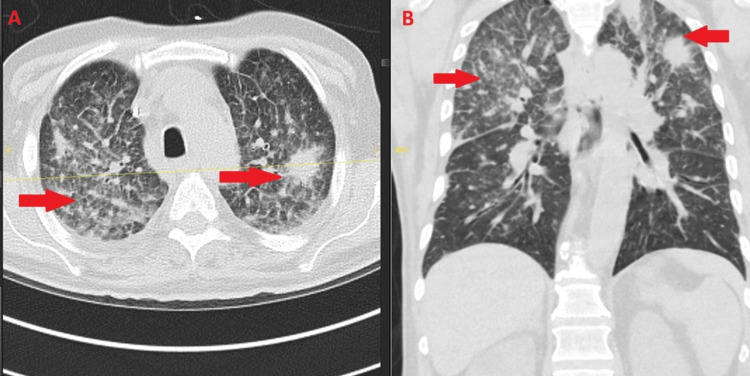
Persistent pulmonary fusariosis after discharge. Axial (A) and coronal (B) chest CT (January 21, 2025) showing persistent bilateral ground-glass and consolidative opacities (arrows), consistent with treated but ongoing pulmonary involvement. CT: computed tomography

At outpatient follow-up on January 17, 2025, the patient reported waxing and waning diarrhea, constipation, and urinary urgency, symptoms felt to be multifactorial and most consistent with treatment-related effects and critical illness rather than progressive fungal infection. His cutaneous lesions demonstrated interval clinical improvement while on fosmanogepix; however, pulmonary disease persisted, with a significant consolidative opacity raising concern for possible superimposed invasive mold infection, such as mucormycosis, although this was never microbiologically confirmed. A follow-up chest CT on January 21, 2025, demonstrated persistent bilateral pulmonary opacities with incomplete radiographic resolution, consistent with ongoing but treated pulmonary fusariosis (Figure 4).

The patient was readmitted on January 23, 2025, for progressive dyspnea and hypoxemia. Blood cultures obtained during this admission grew *Streptococcus mitis*, complicated by severe sepsis with a hyperinflammatory cytokine-mediated response and acute respiratory distress syndrome (ARDS) in the setting of prolonged profound neutropenia. Despite broad-spectrum antimicrobial therapy, his respiratory status continued to deteriorate. After multidisciplinary goals-of-care discussions involving the primary team, Infectious Diseases, and palliative care, the patient elected to pursue comfort-focused measures and was transitioned to hospice care, where he ultimately expired due to complications of secondary bacterial infection in the setting of prolonged immunosuppression.

Case 2

A 62-year-old male with a history of hypertension, type II diabetes mellitus, chronic hepatitis B on entecavir therapy, liver cirrhosis, and pleural effusion was diagnosed with systemic mastocytosis associated with myelodysplastic syndrome (MDS) in December 2024. His disease subsequently progressed to AML with 80% blasts by June 2025, and he presented to the emergency department on October 6, 2025, with two weeks of headache, sinus pressure, and nasal congestion. His hematologic disease had been managed with supportive transfusions of packed red blood cells and platelets, biweekly filgrastim, and treatment with gilteritinib and venetoclax, as intensive cytotoxic chemotherapy was limited by refractory disease, cumulative treatment-related marrow suppression, and significant medical comorbidities. Over the preceding six months, he developed worsening cytopenias attributed to disease progression. He was placed on neutropenic prophylaxis with acyclovir, ciprofloxacin, and posaconazole, while continuing entecavir for chronic hepatitis B suppression. The patient reported a history of recurrent infections, including odontogenic infection, neutropenic colitis, and bilateral pneumonia requiring hospitalization and intravenous antibiotics. He also noted increasing hemoptysis. At presentation, he was receiving ertapenem and vancomycin per Infectious Diseases recommendations for ongoing neutropenic colitis and treatment of an extended-spectrum beta-lactamase (ESBL) *Escherichia coli* liver abscess status post aspiration.

On presentation, laboratory evaluation revealed profound neutropenia with an ANC nadir of approximately 20 cells/µL, which had persisted for several weeks prior to admission, along with severe multilineage cytopenias consistent with advanced bone marrow failure (Table 1). Blood cultures and urinalysis were negative for microbial growth. A CT of the head revealed paranasal sinus disease with opacification of the left ethmoid air cells and near-complete opacification of the left maxillary sinus. Given the patient’s profound neutropenia and sinonasal symptoms, invasive fungal sinusitis was considered in the differential diagnosis. However, otolaryngology consultation and endoscopic sinus evaluation were not pursued due to the absence of focal cranial neuropathies, facial necrosis, or radiographic evidence of bony erosion, as well as the patient’s overall clinical instability and competing sources of DFI. Chest radiograph demonstrated nonspecific hazy ground-glass opacities in the right mid-lung field. A right-sided loculated pleural effusion with a mass-like opacity subsequently required chest tube placement, and Infectious Diseases was consulted for further evaluation.

**Figure 5:**
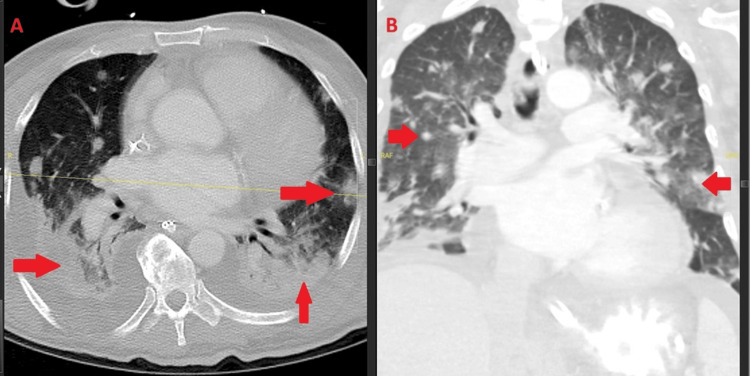
Progressive pulmonary fusariosis. Axial (A) and coronal (B) chest CT (October 31, 2025) showing worsening bilateral nodular and consolidative opacities (arrows). Bronchoalveolar lavage (BAL) confirmed Fusarium solani, consistent with disseminated infection. CT: computed tomography

The patient had a known history of nodular pneumonia since July 2025 that was treated empirically as a presumed fungal infection, based on imaging findings and clinical improvement with posaconazole, without microbiologic confirmation. Posaconazole was continued until radiographic resolution and recovery of the ANC above 500 cells/µL. However, a chest CT on October 31, 2025, demonstrated an interval increase in size and number of bilateral pulmonary nodules with associated consolidative changes, most pronounced in the lower lobes, concerning for progressive invasive mold pneumonia despite prior posaconazole exposure (Figure 5).

**Figure 6:**
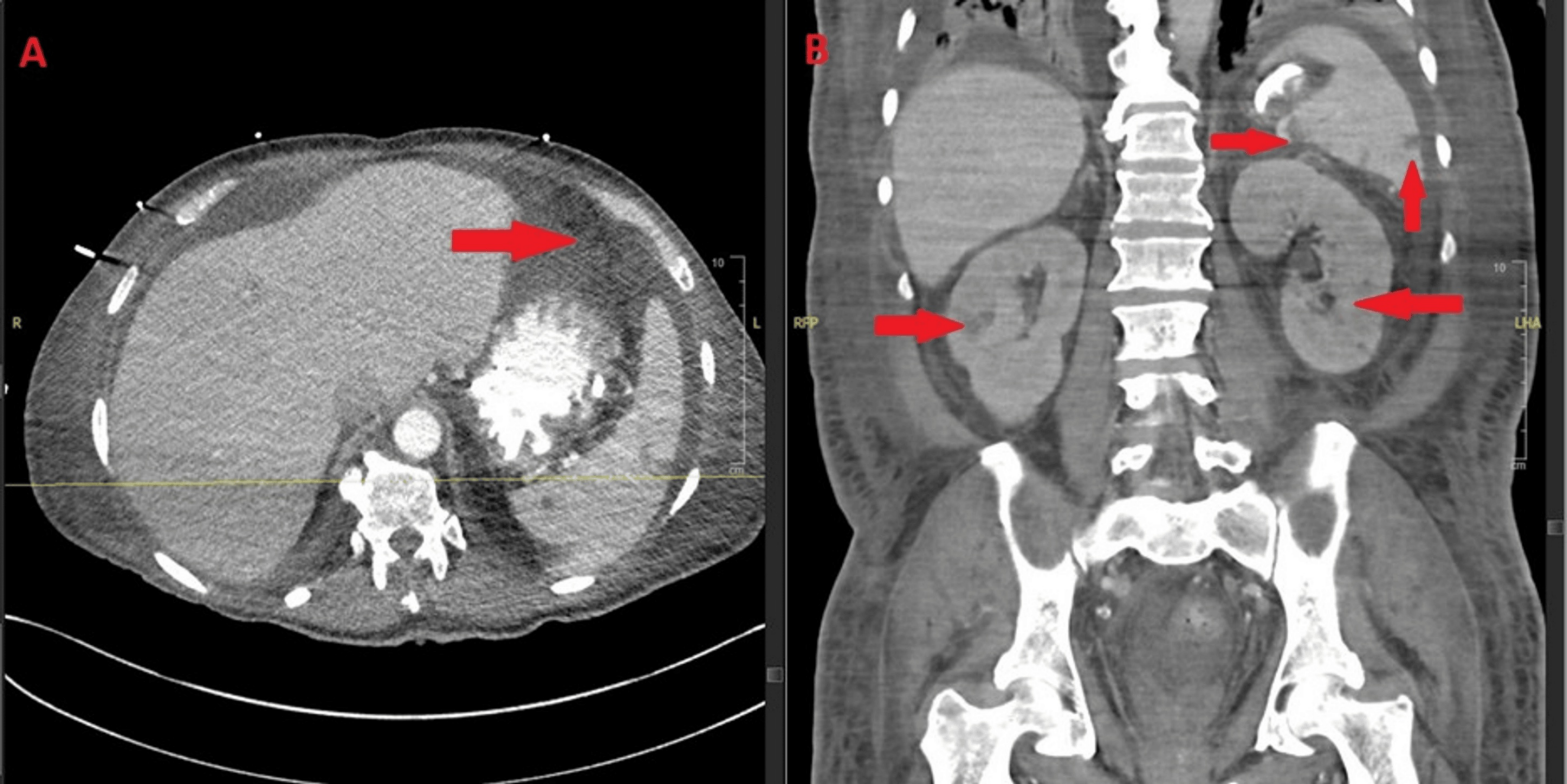
Splenic and renal dissemination in Fusarium solani infection. Axial (A) and coronal (B) abdominal CT (October 31, 2025) showing hypodense splenic and bilateral renal lesions (arrows), consistent with hematogenous dissemination. CT: computed tomography

Further imaging with CT of the abdomen and pelvis on October 31, 2025, revealed hypodense splenic lesions and bilateral renal nodular hypodensities, raising concern for hematogenous fungal dissemination (Figure 6). These findings represented new visceral organ involvement in addition to progressive pulmonary disease.

**Figure 7:**
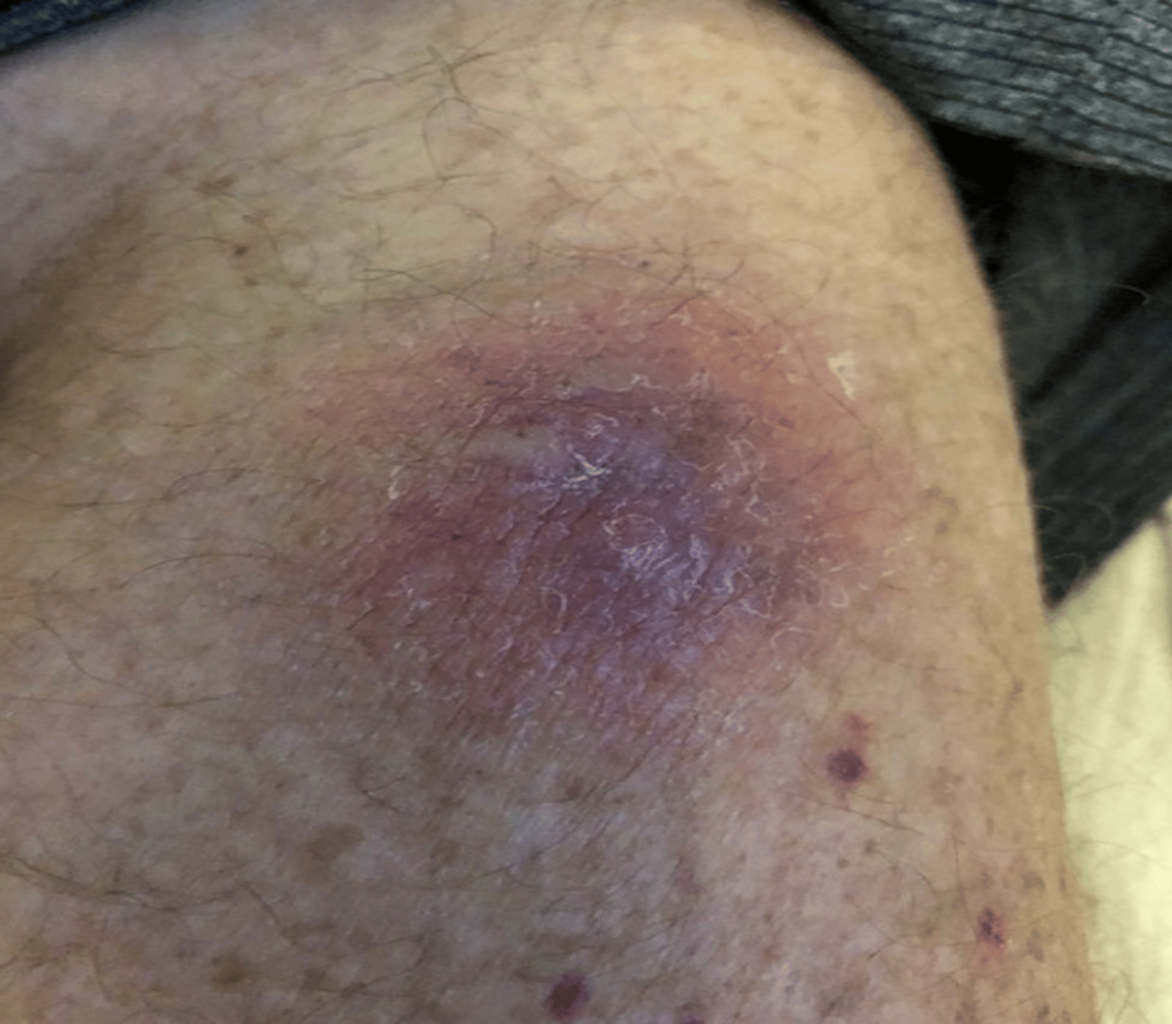
Cutaneous involvement in disseminated Fusarium solani. Clinical photograph showing a violaceous plaque with central duskiness on the left upper extremity; culture confirmed Fusarium solani.

BAL subsequently confirmed *Fusarium solani*, and a culture obtained from a newly developed violaceous erythematous plaque on the left upper extremity also grew *Fusarium*, confirming cutaneous dissemination (Figure 7). Collectively, the pulmonary, splenic, renal, and cutaneous findings established the diagnosis of multisystem disseminated fusariosis.

Given the severity of infection and profound immunosuppression, granulocyte-macrophage colony-stimulating factor (GM-CSF) was initiated to promote neutrophil recovery. Antifungal susceptibility testing demonstrated high-level resistance to azoles and amphotericin B. Despite this, the patient was continued on liposomal amphotericin B (5 mg/kg/day), voriconazole (300 mg twice daily), and terbinafine (125 mg twice daily), and fosmanogepix was requested as salvage therapy. Broad-spectrum antibacterial coverage with meropenem and vancomycin was continued, along with entecavir and prophylactic acyclovir. On November 5, 2025, the patient developed runs of ventricular tachycardia with heart rates exceeding 180 beats per minute and rapidly progressed to asystole, resulting in death. At the time of deterioration, no acute electrolyte abnormalities or documented QT prolongation were identified, and a definitive contribution from antifungal-associated cardiotoxicity could not be established.

Case 3

An 87-year-old female with a history of AML receiving azacitidine and venetoclax for disease control, along with heart failure, hypertension, and prior bilateral hip replacements, was transferred from an outside hospital on October 25, 2025, for further management of AML and concern for fungemia. According to outside hospital records, she had sustained two recent falls with worsening left hip pain. A CT of the pelvis demonstrated a compound fracture of the left inferior pubic ramus. A concurrent CT of the chest revealed an irregular 13-mm pulmonary nodule in the left lower lobe with multiple additional bilateral nodules, with differential considerations including septic emboli versus metastatic disease. Blood cultures obtained at the outside hospital initially demonstrated organisms morphologically consistent with yeast. As a result, she was treated empirically for presumed candidemia with piperacillin-tazobactam and antifungal therapy, initially with voriconazole and subsequently transitioned to micafungin in the absence of definitive fungal biomarker positivity or species-level identification at that time, pending further microbiologic clarification. Several days later, definitive microbiologic identification confirmed that the bloodstream isolate was not a yeast but a filamentous mold identified as *Scedosporium* species.

Upon arrival at our institution, the patient was hypotensive, requiring intravenous fluids and vasopressor support, in atrial fibrillation with rapid ventricular response, intermittently confused, and profoundly neutropenic. This presentation was consistent with severe sepsis and met criteria for an elevated quick sequential organ failure assessment (qSOFA) score (≥2), based on altered mental status and systolic blood pressure (SBP) <100 mmHg (SBP 84 mmHg), reflecting significant acute organ dysfunction and high illness severity at presentation. The laboratory evaluation demonstrated severe neutropenia, anemia, and thrombocytopenia, along with acute kidney injury, transaminitis, and elevated lactate, consistent with evolving multisystem organ dysfunction (Table 1). Infectious Diseases was consulted.

Follow-up communication with the outside hospital revealed that the organism initially reported as yeast on blood cultures was subsequently identified several days later as *Scedosporium prolificans*, a mold associated with rapid dissemination and intrinsic resistance to most antifungal agents. This delay in definitive organism identification contributed to initial antifungal misclassification and delayed initiation of appropriate mold-active therapy. Antifungal therapy was promptly adjusted to voriconazole and terbinafine, and piperacillin-tazobactam and micafungin were discontinued. A plasma-based Karius test performed at our institution also detected *Scedosporium prolificans*, confirming the organism as the cause of fungemia.

**Figure 8:**
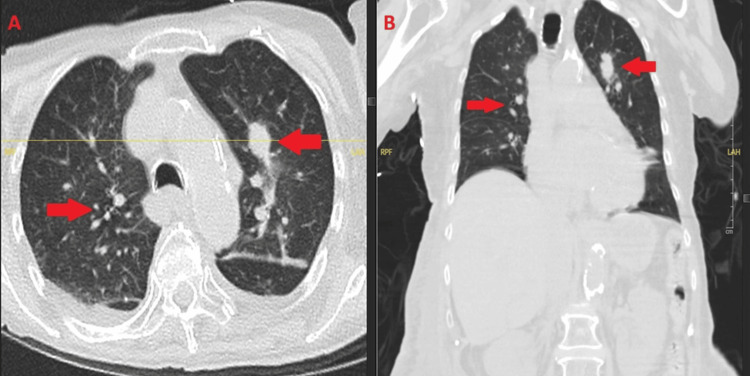
A chest CT scan obtained during hospitalization showed pulmonary involvement in Scedosporium prolificans fungemia. Axial (A) and coronal (B) chest CT showing multiple bilateral pulmonary nodules (arrows), consistent with hematogenous dissemination. CT: computed tomography

CT imaging of the chest, abdomen, and pelvis obtained during hospitalization demonstrated bilateral nodular airspace opacities that were stable to minimally worsened compared with prior imaging, favoring an infectious or inflammatory etiology in the setting of fungemia (Figure 8). Given the known neurotropism of *Scedosporium prolificans*, CNS involvement was concurrently evaluated, as detailed below. No additional acute intra-abdominal or pelvic source of infection was identified.

**Figure 9:**
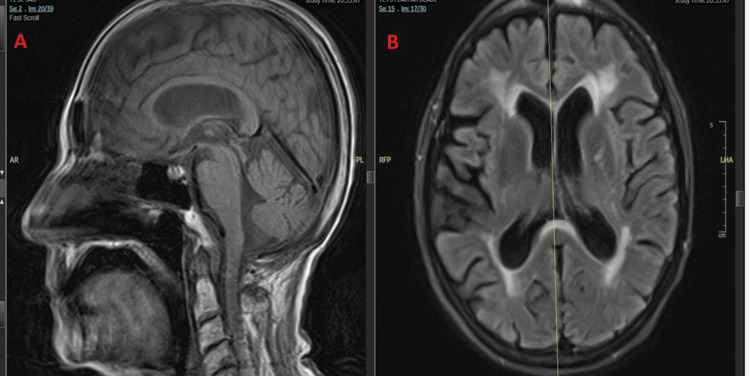
Brain MRI in Scedosporium prolificans fungemia. Sagittal T1 (A) and axial FLAIR (B) images showing no acute intracranial lesion; early or radiographically occult CNS involvement cannot be excluded. MRI: magnetic resonance imaging; FLAIR: fluid-attenuated inversion recovery; CNS: central nervous system

According to the patient’s son, she had experienced progressive confusion over the preceding week, whereas she had been living independently at baseline. Given concern for toxic-metabolic encephalopathy versus early CNS involvement in the setting of bloodstream infection with *Scedosporium prolificans*, magnetic resonance imaging (MRI) of the brain was obtained. The examination was significantly limited by motion artifact and performed without intravenous contrast, and no acute intracranial lesion or abnormal enhancement was identified (Figure 9). However, in the context of proven mold fungemia, enlarging pulmonary nodules, profound immunosuppression, and progressive encephalopathy, radiographically occult or early CNS dissemination remained a clinical concern, as CNS involvement may precede detectable imaging findings in angioinvasive mold infections. Lumbar puncture was not pursued due to the patient’s hemodynamic instability and inability to tolerate the procedure.

Despite antifungal therapy and supportive care, the patient’s clinical status continued to deteriorate with evidence of multisystem organ dysfunction. Given her advanced age, profound immunosuppression, refractory fungemia, and poor overall prognosis, goals-of-care discussions were held with the family. Ultimately, the decision was made to pursue comfort-focused care, and the patient was transitioned to hospice.

The pertinent laboratory abnormalities observed at presentation across the three cases are summarized in Table 1. The clinical characteristics, microbiologic findings, treatment courses, and outcomes of the three cases are summarized in Table 2.

**Table 2:** Clinical characteristics and outcomes of the three patients with DFIs. DFI: disseminated fungal infection; MDS: myelodysplastic syndrome; AML: acute myeloid leukemia; CNS: central nervous system; *S. mitis*: *Streptococcus mitis*; ARDS: acute respiratory distress syndrome

Characteristic	Case 1	Case 2	Case 3
Age/sex	75-year-old male	62-year-old male	87-year-old female
Underlying hematologic disease	Trisomy 8-associated AML	Systemic mastocytosis with MDS → AML	AML
Absolute neutrophil count at presentation	0 cells/µL	20 cells/µL	approximately 200 cells/µL
Initial site(s) of infection	Skin, lungs	Lungs	Bloodstream, lungs
Identified pathogen	Fusarium species	Fusarium solani	Scedosporium prolificans
Evidence of dissemination	Skin, lungs	Lungs, spleen, kidneys, skin	Bloodstream, lungs (possible CNS)
Antifungal therapy	Amphotericin B, voriconazole, terbinafine → fosmanogepix	Amphotericin B, voriconazole, terbinafine; fosmanogepix requested	Voriconazole + terbinafine
Clinical course	Cutaneous improvement; persistent pulmonary disease	Rapid progression with multisystem dissemination	Progressive encephalopathy and organ failure
Outcome	Death due to secondary bacterial infection (S. mitis, ARDS)	Sudden cardiac death	Transitioned to hospice

## Discussion

DFI remains one of the most devastating infectious complications in immunocompromised patients, as illustrated by the three cases presented in this series and supported by prior cohort data. In a large series of invasive fusariosis, disseminated disease has been reported in approximately 67-72% of cases, with a markedly higher incidence among neutropenic patients compared with non-neutropenic hosts, underscoring the central role of host immune failure in disease progression [5]. Despite differences in underlying hematologic disease and fungal pathogens, all patients in this series shared key risk factors for dissemination, including profound immune dysfunction and impaired innate host defenses. Across Cases 1 and 2, disseminated fusariosis manifested with pulmonary nodules and characteristic cutaneous lesions, whereas Case 3 demonstrated bloodstream infection with pulmonary involvement and suspected CNS dissemination. Collectively, these cases highlight that DFI often presents with overlapping clinical patterns and that outcomes are largely determined by the degree and reversibility of immune suppression rather than by antifungal therapy alone.

The central role of neutrophils in preventing fungal dissemination is underscored by the clinical courses observed in all three patients. Neutrophils serve as the primary effector cells of antifungal innate immunity through phagocytosis, oxidative burst-mediated killing, release of antimicrobial peptides, and formation of neutrophil extracellular traps that damage invasive hyphae [10]. Beyond direct pathogen clearance, neutrophils coordinate downstream immune responses by recruiting monocytes, dendritic cells, and T lymphocytes, thereby linking innate and adaptive immunity. T helper cell responses and antibody-mediated opsonization further augment fungal containment [3]. In the absence of adequate neutrophil number or function, fungal conidia can germinate into angioinvasive hyphal forms, penetrate vascular endothelium, and disseminate hematogenously, resulting in tissue infarction and multiorgan involvement.

Importantly, all three patients in this series experienced prolonged, profound neutropenia driven by refractory leukemia and bone marrow aplasia resulting from ongoing disease-control strategies rather than curative therapy. In Case 1, ANCs remained near zero for approximately 2-3 months before and throughout the diagnosis of disseminated infection. Case 2 experienced at least 6-8 weeks of persistent severe neutropenia in the setting of progressive AML and marrow failure, while Case 3 had several weeks of unresolved neutropenia prior to presentation and during hospitalization. Across cases, ANCs were persistently suppressed, generally ranging from 0 to approximately 200 cells/µL. The increasing use of newer leukemia regimens such as azacitidine-venetoclax has prolonged survival in patients with otherwise incurable disease, but at the cost of sustained marrow suppression and extended vulnerability to opportunistic infections. In addition, prolonged transfusion dependence in this setting, most notably in Case 1, who required frequent red blood cell and platelet transfusions during hospitalization, may result in secondary iron overload, creating a permissive microenvironment for certain angioinvasive molds. In this series, iron availability may have further amplified susceptibility to disseminated fusariosis, whereas its role in *Scedosporium prolificans* infection remains less well defined. Collectively, these factors illustrate how modern leukemia management can paradoxically extend life while simultaneously amplifying the biologic conditions that favor fungal dissemination and poor outcomes.

Pathogen-specific virulence factors further contribute to dissemination and immune evasion in DFI, particularly when host immune containment is lost. In disseminated candidiasis, hyphal transformation facilitates tissue invasion, while biofilm formation protects organisms from host defenses and antifungal agents by sequestering drugs within the extracellular matrix [2]. In Case 3, *Scedosporium prolificans* demonstrated several of these aggressive traits, including transition from conidia to invasive hyphae, vascular invasion, and biofilm formation, features that are associated with rapid dissemination, fungemia, and resistance to immune clearance [3]. Melanin production represents an additional virulence factor relevant to several molds, including *Scedosporium* species, protecting against oxidative stress and modulating host immune responses by inhibiting phagolysosome formation and host cell apoptosis [4]. Some fungi evade complement-mediated immunity by recruiting host plasminogen, which cleaves the complement component C3 [10], while others, including *Candida glabrata* and *Histoplasma capsulatum*, can survive intracellularly following phagocytosis.

In addition to intrinsic virulence mechanisms, the route and chronicity of exposure may influence dissemination once immune defenses fail. In Cases 1 and 2, prolonged neutropenia likely permitted transition from localized or superficial infection to invasive disease; chronic colonization or infection of the skin or nails, such as onychomycosis, paronychia, or interdigital skin colonization, has been described as a potential portal of entry for *Fusarium* species and may persist for months or years prior to invasive infection [13]. In the absence of local containment, angioinvasive molds such as *Fusarium* spp., as observed in Cases 1 and 2, promote vascular thrombosis, tissue ischemia, and necrosis, contributing to fungemia and the characteristic necrotic cutaneous lesions seen in disseminated fusariosis [5,8,9]. Additional immune evasion mechanisms described in other pathogenic fungi, including the hydrophobin coat of *Aspergillus fumigatus*, the large polysaccharide capsule and intracellular escape of *Cryptococcus neoformans*, and spherule formation by *Coccidioides immitis, *further illustrate conserved strategies by which fungi circumvent host immune responses in the setting of profound immunosuppression [10].

Diagnosis of DFI remains challenging and was delayed in a variable but clinically meaningful manner across the cases presented, typically by days to weeks from initial presentation. In Case 1, although cutaneous lesions were present early, confirmation of disseminated fusariosis required a repeat biopsy and culture after transfer of care, contributing to diagnostic delay. In Case 2, progression from initially responsive pulmonary disease to multisystem dissemination occurred despite prior mold-active prophylaxis, with definitive diagnosis established only after BAL and tissue cultures during clinical deterioration. In Case 3, early blood cultures initially suggested yeast, delaying recognition of filamentous mold fungemia until definitive identification of *Scedosporium prolificans* and confirmatory plasma microbial cell-free DNA testing were obtained. These delays reflect inherent limitations of current diagnostic modalities. Conventional cultures may be slow or insensitive, particularly for molds, and may be confounded by contamination or cross-reactivity [14]. Some fungi grow poorly or not at all in routine culture systems, while others require prolonged incubation. Although *Fusarium* and *Scedosporium* are notable exceptions because they can cause fungemia and be recovered from blood cultures, as demonstrated in Case 3, culture-based diagnosis alone is often insufficient. In Cases 1 and 2, histopathologic examination and culture of skin lesions, along with BAL cultures, were ultimately critical for diagnosis [3]. Non-culture-based assays, such as galactomannan and β-D-glucan, may provide supportive evidence but lack sensitivity and specificity for certain molds; serum β-D-glucan demonstrates approximately 80% sensitivity for invasive candidiasis but lower specificity (64%), with false-positive rates as high as 75% in patients with concurrent bacteremia, hemodialysis exposure, or intravenous immunoglobulin administration [2]. Advanced molecular diagnostics, including matrix-assisted laser desorption/ionization-time-of-flight (MALDI-TOF) mass spectrometry, polymerase chain reaction (PCR), ribosomal ribonucleic acid (rRNA), internal transcribed spacer (ITS) sequencing, and plasma microbial cell-free DNA testing, may facilitate earlier pathogen identification when conventional methods are inconclusive [8,14,15]. Importantly, radiographic findings may lag behind clinical disease, particularly in CNS involvement, as illustrated by Case 3, in which progressive encephalopathy occurred despite initially non-diagnostic neuroimaging.

Management of DFI is complex and, in the cases presented, was limited by antifungal resistance, drug toxicity, restricted spectrum of activity, and failure of immune recovery [1]. In patients with hematologic malignancies, systemic antifungal prophylaxis reduces the incidence of DFI by approximately 31%, and mold-active prophylaxis with itraconazole, voriconazole, or posaconazole reduces DFI-related mortality, with posaconazole demonstrating the most significant protective effect in patients with prolonged neutropenia [3]. Despite prophylaxis, a breakthrough disseminated infection occurred in this series, highlighting the limitations of current preventive strategies in profoundly immunocompromised hosts. Combination antifungal therapy is frequently employed; however, outcomes remain poor in the absence of neutrophil recovery. In Cases 1 and 2, azole therapy was administered in accordance with institutional standards; however, routine therapeutic drug monitoring was not consistently available or feasible during periods of clinical instability, and subtherapeutic exposure cannot be fully excluded as a contributing factor to disease progression. Recommended treatment for fusariosis includes voriconazole or amphotericin B as initial therapy and posaconazole as salvage therapy [8]. Emerging agents such as olorofim, an orotomide that inhibits pyrimidine biosynthesis, demonstrate activity against molds, including *Aspergillus*, *Scedosporium/Lomentospora*, and *Scopulariopsis* spp., as well as dimorphic fungi [16]. Fosmanogepix, which inhibits glycosylphosphatidylinositol-anchored wall transfer protein (Gwt1), required for biofilm and germ tube formation, showed partial clinical benefit in one case and offers additional advantages, including oral and intravenous formulations and CNS penetration [1,16,18]. Increasing attention has also focused on the fungal-infection microenvironment, including biofilms, site-specific physiologic conditions, and interactions with symbiotic bacteria, which may influence therapeutic responses [16]. Ultimately, antifungal therapy alone is insufficient when immune dysfunction persists; adjunctive immune-restoration strategies, including G-CSF or GM-CSF administration [9], granulocyte transfusion [19], and surgical source control when feasible [5], remain critical determinants of outcome.

Ultimately, these cases highlight the aggressive nature of DFI in immunocompromised hosts and the limitations of current diagnostic and therapeutic strategies. Even when fungal disease appears partially controlled, as in Case 1, patients remain highly vulnerable to secondary infections and rapid clinical deterioration. These observations emphasize the need for early recognition, multidisciplinary management, and timely discussions of goals-of-care in patients with advanced immunosuppression.

This case series has several important limitations that merit consideration. The small sample size and retrospective design limit generalizability, as these cases reflect severe presentations in a highly selected population of profoundly immunocompromised patients. Microbiologic confirmation of dissemination varies across cases and organ systems. In particular, in Case 3, although *Scedosporium prolificans* fungemia and progressive pulmonary disease were clearly documented, CNS dissemination could not be definitively confirmed. Lumbar puncture and cerebrospinal fluid studies were not performed due to hemodynamic instability, and brain MRI was limited by motion artifact and lack of contrast enhancement. As such, CNS involvement was inferred from clinical deterioration, progressive encephalopathy, and the known neurotropism and angioinvasive potential of *Scedosporium* species, rather than from direct laboratory confirmation. Additionally, antifungal susceptibility testing and access to novel agents such as fosmanogepix were not uniform across cases, reflecting real-world constraints that may have influenced treatment decisions and outcomes. Despite these limitations, the cases collectively illustrate consistent clinical patterns of DFI in immunocompromised hosts and underscore the diagnostic and therapeutic challenges encountered in this high-risk population.

## Conclusions

DFI remains a devastating complication in immunocompromised patients with prolonged neutropenia. Across the three cases presented, common patterns of dissemination, including pulmonary nodules, cutaneous lesions, and fungemia, were observed despite differing fungal pathogens and immunosuppressive contexts. Outcomes were uniformly poor, reinforcing that prognosis is primarily driven by the severity and reversibility of host immune dysfunction rather than by antifungal therapy alone. These cases further underscore persistent diagnostic challenges, including nonspecific imaging findings and delayed or incomplete microbiologic confirmation, and highlight the importance of heightened clinical suspicion when pulmonary and cutaneous manifestations coexist in profoundly neutropenic patients, even with antifungal prophylaxis. Although emerging antifungal agents may expand treatment options for resistant molds, immune recovery remains the cornerstone of successful management. Ultimately, DFI in immunocompromised hosts requires early multidisciplinary involvement, aggressive diagnostic evaluation, integration of antifungal therapy with strategies aimed at immune restoration, and timely goals-of-care discussions, particularly in patients with advanced disease and limited potential for immune recovery.
